# Hormonal Therapy Patterns in Older Men with Prostate Cancer in the United States, 2010–2019

**DOI:** 10.3390/cancers17193231

**Published:** 2025-10-04

**Authors:** Mohanad Albayyaa, Yong-Fang Kuo, Vahakn Shahinian, David S. Lopez, Biai Digbeu, Randall Urban, Jacques Baillargeon

**Affiliations:** 1 Department of Cardiovascular Medicine, John Sealy School of Medicine, University of Texas Medical Branch, Galveston, TX 77555, USA; 2 Department of Biostatistics and Data Science, School of Public and Population Health, University of Texas Medical Branch, Galveston, TX 77555, USA; 3 Department of Internal Medicine, School of Medicine, University of Michigan, Ann Arbor, MI 48104, USA; 4 Department of Epidemiology, O’Donnell School of Public Health, University of Texas Southwestern Medical Center, Dallas, TX 75390, USA; 5 Harold Simmons Comprehensive Cancer Center, University of Texas Southwestern Medical Center, Dallas, TX 75390, USA; 6 Department of Internal Medicine, John Sealy School of Medicine, University of Texas Medical Branch, Galveston, TX 77555, USA; 7 Department of Public Health, Robbins College of Health and Human Sciences, Baylor University, Waco, TX 76798, USA

**Keywords:** hormonal therapy, GnRH agonists, GnRH antagonists, anti-androgens, orchiectomy, prostate cancer

## Abstract

**Simple Summary:**

Hormonal therapy is a mainstay treatment for men with prostate cancer, especially those with advanced disease. However, how and when it is used has changed over time, and understanding these changes is important to ensure that patients receive the most appropriate care. In this study, we examined national data on older men with prostate cancer between 2010 and 2019 to see how the use of hormonal therapy has shifted. We found that use of hormonal therapy after diagnosis of higher-risk prostate cancer has steadily increased, while use of hormonal therapy as the only treatment in lower-risk cases has highlighted persistent inappropriate use. These findings provide important insight into how treatment choices have evolved and highlight opportunities to improve care by limiting inappropriate treatment and ensuring that men who may benefit most are receiving it.

**Abstract:**

Importance: Understanding trends in the use of hormonal therapy (HT) for prostate cancer (PCa) is crucial to optimize treatment strategies, particularly for older men with locally advanced and metastatic disease. Objective: To evaluate changes in the patterns of adjuvant and primary HT use over time in older U.S. men diagnosed with locally advanced and metastatic prostate cancer. Design, Setting, and Participants: This cohort study utilized SEER-Medicare data, which covers approximately 48% of the U.S. population and links cancer registry data with Medicare claims, including 149,515 men aged ≥66 years diagnosed with PCa between 2010 and 2019. We analyzed trends in the use of adjuvant HT for higher-risk and primary HT for lower-risk PCa. Multivariable logistic regression models were used to adjust for clinical and demographic factors. Main Outcomes and Measures: The primary outcome was the proportion of men receiving any form of HT within 6 months of PCa diagnosis. HT included injectable Gonadotropin-releasing hormone (GnRH) agonists and antagonists, orchiectomy, and anti-androgens agents. Results: The rate of adjuvant HT in higher-risk PCa patients increased significantly from 53.6% in 2010 to 68.1% in 2019 (*p* < 0.0001), with a steady rise in the last four years. In contrast, the rate of men with lower-risk disease receiving primary HT declined from 25% in 2010 to 16.9% in 2013, then peaked at 28.2% in 2015, and stabilized between 25% and 27.3% from 2017 to 2019. The overall HT usage increased from 33.5% in 2010 to 45.2% in 2019, showing a consistent increase over the years. These patterns persisted after adjusting for clinical and demographic factors. Conclusions and Relevance: The increasing use of adjuvant HT in higher-risk PCa patients aligns with evolving treatment guidelines, while the stable rate of primary HT in lower-risk patients represents persistent inappropriate use and highlights the need for further efforts to optimize treatment choices. While previous studies focused on men with intermediate-risk PCa receiving radiation therapy, our study broadens the scope to include men who did not undergo radiation therapy, providing a more inclusive view of HT trends. Future research should focus on refining strategies to reduce inappropriate primary HT use and improve adjuvant HT administration.

## 1. Introduction

Prostate cancer (PCa) is the most common nonskin malignancy among men, representing a significant health burden [1,2,3]. Hormonal therapy (HT) includes both androgen deprivation therapy (ADT)—such as gonadotropin-releasing hormone (GnRH) agonists, GnRH antagonists, and orchiectomy—and anti-androgens. In recent years, anti-androgens have played a significant role when combined with GnRH agonists in the treatment of metastatic, castration-sensitive, and castration-resistant prostate cancer (mCRPC) [4,5,6,7,8]. Despite HT’s long-standing use, clinical trial data supporting the survival benefits of HT as a standalone treatment for locally advanced PCa remain limited [7,8].

The current National Comprehensive Cancer Network (NCCN) guidelines recommend HT for PCa patients, often in combination with radiation therapy, due to its demonstrated benefits in improving overall survival and reducing the risk of metastasis [9,10]. By 2016, the guidelines evolved further, incorporating recommendations for the use of GnRH agonists in combination with newer anti-androgen agents, such as abiraterone acetate and enzalutamide, particularly for the treatment of mCRPC [11,12,13,14]. Subsequent revisions in 2018 and 2020 have focused on refining recommendations regarding HT duration based on various clinical factors, such as disease stage, risk stratification, and patient preferences [15,16,17]. These changes have influenced real-world practice patterns, particularly the growing adoption of novel agents in advanced disease [16].

For patients with early-stage and low-grade PCa, HT is often considered inappropriate due to minimal or nonexistent survival benefits and the potential for significant adverse effects, including decreased bone density, increased cardiovascular risk, diabetes, obesity and diminished quality of life [18,19]. HT is generally not recommended for patients who have had a prostatectomy unless there is evidence of recurrent or metastatic PCa [20,21]. Persistent and inappropriate use of primary HT in low-risk patients raises concerns about overtreatment, especially among older men with competing comorbidities [22].

The rising number of PCa diagnoses in the U.S., driven by an aging population and improvements in diagnostic tools and screening, may also contribute to the observed increase in overall HT use [21,22]. Understanding whether these increases reflect guideline-concordant care, however, requires analyses that distinguish between adjuvant and primary HT use. Over the last decade, research has highlighted notable shifts in HT use across patient risk groups and clinical settings [20,21,22,23]. While previous research has explored shifts in ADT patterns, particularly among patients with low-risk PCa, few studies often emphasize general usage trends without examining the ADT use as primary or adjuvant therapy in relation to clinical guidelines or differentiating by specific patient risk groups [21,22]. In addition, no published studies have specifically reported on the pattern of anti-androgen use, particularly in combination with other ADT agents, using a national population-based data source. Few studies have provided focused analyses of ADT usage patterns, particularly among patients receiving radiation therapy and without including anti-androgen agents or examining their role in such trends [22]. As the first study to address this gap, our findings provide valuable insights into prescribing trends and their relevance to clinical practice.

The objective of our study is to address gaps in understanding primary and adjuvant HT usage by assessing HT patterns, including both ADT and anti-androgens, among older US men diagnosed with locally advanced and metastatic PCa between 2010 and 2019 using nationally representative the Surveillance, Epidemiology, and End Results (SEER)-Medicare database [23]. This study extends analyses through 2019 and assess both primary and adjuvant HT, offering a more comprehensive assessment of national trends and their alignment with evolving treatment guidelines, while also highlighting areas of persistent inappropriate use. Our analysis represents a large-scale, national assessment of HT trends over time, extending four years beyond the most recent study and providing a more current and comprehensive analysis of HT patterns than prior research.

## 2. Methods

### 2.1. Data Sources

Data for this study were obtained from the linked SEER-Medicare national real-world database. The SEER program collects cancer incidence, treatment, and survival data from various cancer registries covering approximately 48% of the US population. It includes information on patient demographic factors, tumor characteristics, treatments received, and outcomes from 17 population-based tumor registries [24]. Medicare, a federal health insurance program in the United States, primarily provides health coverage for 97% of individuals aged ≥65 [23,24].

### 2.2. Study Subjects

The study included patients aged ≥66 years listed in the SEER-Medicare database who received their initial diagnosis of PCa between 2010 and 2019, totaling 548,635 subjects. To be included in the study cohort, patients were required to have been enrolled in Medicare Part A, B, and D for a minimum of 12 months prior to and 6 months after their PCa diagnosis date, and to have not received care from a Health Maintenance Organization within 12 months prior PCa diagnosis (see Appendix A). This resulted in 149,515 patients for primary analyses. Patients who had undergone prostatectomy were excluded.

Patients who had undergone prostatectomy were excluded to minimize misclassification of adjuvant versus salvage HT, which cannot be reliably distinguished in SEER-Medicare claims data. Because men undergoing prostatectomy may later receive HT for biochemical recurrence or metastatic progression, distinguishing these settings is challenging; thus, exclusion ensures cleaner categorization [25,26]. SEER-Medicare data included various aspects of clinical characteristics such as cancer stage, cancer grade, Charlson comorbidity score, concomitant medications, primary HT treatments, and patient demographics such as age, race, education, marital status, region, and year of diagnosis.

### 2.3. Indications for HT

We categorized the patients into two groups based on stage, grade, and receipt of radiation therapy within 6 months after PCa diagnosis, where treatment intent could be most reliably inferred from claims.

Higher-risk disease: This category included patients who have been diagnosed at stage (T3 or T4) with a high grade (poorly differentiated or undifferentiated) and had received radiation therapy within 6 months after PCa diagnosis. Patients with metastatic PCa and received radiation therapy were included in this group, reflecting contemporary guideline-based management of advanced disease.

Lower-risk disease: This category included patients who have been diagnosed with localized stages (T1 or T2) with a low grade (well-differentiated or moderately differentiated) and have not received radiation therapy.

This classification was designed to align with guideline-based indications, allowing to examine appropriate (adjuvant HT in higher-risk disease treated with radiation) versus inappropriate use (primary HT in lower-risk disease without radiation). Patients with high-risk disease who did not receive radiation were excluded. This approach reduced potential misclassification and focused the analysis on groups where treatment intent could be most clearly inferred. For each of these groups, we examined the trends in the use of HT, different types—GnRH agonists, antagonists, orchiectomy, and anti-androgens—by calendar year. Although orchiectomy was examined, the sample size was too small to report.

### 2.4. Definitions

For the cohort of men who received a diagnosis of incident PCa from 2010 to 2019, we examined the proportion of PCa patients who received any form of HT in the 6 months following PCa diagnosis. HT included injectable GnRH agonists and antagonists (covered under Part B), orchiectomy, and anti-androgens agents (covered under Part D), based on Healthcare Common Procedure Coding System (HCPCS) drug administration and National Drug Code (NDC) codes. Anti-androgens were categorized into first-generation agents (bicalutamide, flutamide, nilutamide) and novel agents (abiraterone acetate, enzalutamide) using NDCs. Radiation therapy (including External beam radiation therapy (EBRT), Brachytherapy, and seed implantation) and orchiectomy were identified using Current Procedural Terminology (CPT) codes and International Classification of Diseases (ICD 9 and ICD 10) codes (Appendix B) [27]. Comorbidity was evaluated using Klabunde’s adaptation of the Charlson comorbidity index, based on Medicare claims data from the year before the PCa diagnosis [28,29].

### 2.5. Statistical Analyses

Descriptive analysis was conducted to assess demographic and clinical characteristics in all patients, including those with higher-risk disease and those with lower-risk disease. The Cochran-Armitage test was used to assess trends of HT usage over time. Multivariable logistic regression analyses were used to examine the extent to which calendar year, adjusting for demographic and clinical factors, was associated with HT use. Calendar year was modeled as a categorical variable (reference = 2010), so odds ratios represent the odds of HT use in each year compared with 2010, All statistical tests were two-sided and considered significant at an alpha level of 0.05. Statistical analyses were conducted using SAS software (RRID:SCR_008567), version 9.4 (SAS Institute Inc., Cary, NC, USA) [30]. The study protocol received approval from the University of Texas Medical Branch (UTMB) institutional review board [31].

### 2.6. Data Availability

The datasets used to conduct this study are available upon approval of a research protocol from the National Cancer Institute. Instructions for obtaining these data are available at https://healthcaredelivery.cancer.gov/seermedicare/obtain/ (accessed on 7 January 2024).

## 3. Results

### 3.1. Patient and Cancer Characteristics

Table 1 presents: the rates of hormonal therapy (HT) use among men with newly diagnosed prostate cancer (PCa) (*n* = 149,515); the rates of adjuvant HT use among men with higher-risk PCa (*n* = 6769); and the rates of primary HT use among men with lower-risk PCa (*n* = 73,840).

The overall rate of HT use increased steadily from 31.6% in 2010 to 40.2% in 2019, an increase of 8.6 percentage points The use of adjuvant HT was most prevalent in advanced disease stages, with 59.1% of Stage III and 55.9% of Stage IV patients receiving it. Tumor differentiation also influenced HT use: 73.8% of men with poorly differentiated tumors received adjuvant HT, compared to 23.0% with moderately differentiated tumors and 4.9% with well-differentiated tumors (*p* < 0.0001). Comorbidity burden further correlated with HT utilization; men with a Charlson Comorbidity Index (CCI) score of ≥3 had the highest rate of any HT use (38.5%), while those with a score of 0 had the lowest (31.4%) (*p* < 0.0001).

Age was strongly associated with HT use, with the highest rates observed in men aged ≥80 (43.4% receiving any HT). Primary HT use peaked in men aged ≥80 (33.8%) and was lowest in men aged 66–70 (16.8%) (*p* < 0.0001). Racial disparities were also evident: Hispanic men had the highest overall HT utilization (40.5%), followed by black (38.1%) and white men (34.2%) (*p* < 0.0001). These disparities persisted after adjustment for age and comorbidity, suggesting that geographic clustering, barriers to specialty urologic care and socioeconomic factors may contribute to higher HT use among Hispanic men.

**Figure 1:**
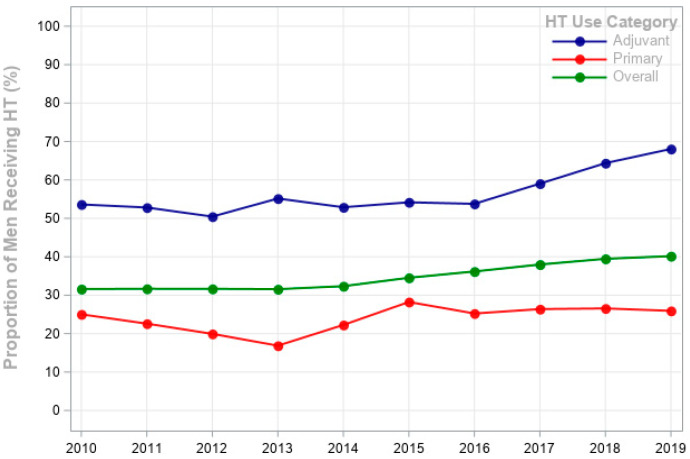
Annual Rate of Use of Hormonal Therapy (HT) within 6 Months after the Diagnosis of Prostate Cancer (PCa) from 2010 through 2019. Shown are the proportions of men receiving overall HT use (at least one dose of a gonadotropin-releasing hormone agonist, antagonist, or anti-androgen). Adjuvant use includes patients who underwent radiation therapy for the treatment of clinical stage T3 or T4 tumors and high grade (poorly differentiated or undifferentiated). Primary use includes those who had clinical stage T1 or T2 tumors, low grade (well-differentiated or moderately differentiated) and did not undergo radiation therapy.

In Figure 1, the proportion of men receiving HT within 6 months after diagnosis for both adjuvant and primary groups is shown over time. The figure shows an upward trend in adjuvant HT use from 53.6% in 2010 to 68.1% in 2019 (*p* < 0.0001), demonstrating a steady increase in its overall use. The proportion of men with lower-risk disease receiving primary HT declined from 25% in 2010 to 16.9% in 2013, then peaked at 28.2% in 2015, and stabilized between 25% and 27.3% from 2017 to 2019 (*p* = 0.084). Although guidelines do not recommend primary HT in low-risk PCa, approximately one in four men with low-risk PCa continued to receive primary HT throughout the study period, indicating persistent inappropriate use. The overall HT usage increased from 33.5% in 2010 to 45.2% in 2019, showing a consistent increase over the years.

Table 2 presents trends in HT use for each specific HT category. In the adjuvant HT, GnRH agonist use increased from 8.9% in 2010 to 13.6% in 2019, while GnRH antagonist use rose significantly from 2.5% in 2010 to 20.2% by 2019. Anti-androgen increased from 8.0% in 2010 to 15.3% by 2019. In the primary HT, GnRH agonist use initially declined from 8.6% in 2010 to 5.6% in 2013, peaked at 13.3% in 2015, and then stabilized around 12%. GnRH antagonist use increased significantly from 3.6% in 2010 to 15.8% by 2015, then stabilized around 14%. While anti-androgen declined from 7.9% to 5.9% in 2015, then increased to 13.1% in 2019. In clinical practice, anti-androgens are often prescribed in combination with GnRH agonists or antagonists. However, in our analysis we categorized patients according to the specific HT they received, and each patient was assigned to only one category (GnRH agonist, GnRH antagonist, or anti-androgen).

### 3.2. Multivariable Analysis

Table 3 presents the results of the multivariable logistic regression predicting HT use by calendar year. Calendar year was modeled as a categorical variable (reference year: 2010), and odds ratios represent the odds of HT use in each year compared with 2010. Absolute percentages were also reported alongside ORs to improve interpretability. The adjusted odds of overall HT use increased over the study period, with a monotonic increase from 2015 to 2019, OR of 1.45 (95% CI: 1.38–1.52) for the comparison of 2019 to 2010. In the cohort for whom adjuvant HT was used for patients with higher-risk PCa, HT use showed progressively higher odds in later years compared with 2010, with the highest increase observed in 2019 (OR 1.84, 95% CI: 1.46–2.33). In the cohort for whom primary HT was used for lower-risk PCa, the odds of receiving HT fluctuated over the years, peaking in 2015 with an OR of 1.17 (95% CI: 1.08–1.28) but stabilizing thereafter, showing no significant change from 2010. This pattern highlights that while adjuvant HT use expanded in accordance with evolving guidelines, primary HT use inappropriately persisted in low-risk patients without meaningful decline over time.

## 4. Discussion

This study presents an analysis of HT, which includes ADT and anti-androgen agents, patterns among PCa patients in the United Sates from 2010 to 2019. We observed an increase, over the study period, in overall HT use, and increase from 2016 to 2019 for adjuvant HT in patients with higher-risk disease. In contrast, primary HT use showed an inconsistent pattern over time, ranging between a low point of 16.9% in 2013 and a high point of 28.2% in 2015. Notably, approximately one in four men with low-risk PCa continued to receive primary HT in 2019, underscoring persistent inappropriate use. Although demographic aging and advances in diagnostic pathways could partly explain increases in HT use, the significant association between calendar year and HT persisted after multivariable adjustment for age, comorbidity, and tumor characteristics. This suggests that evolving guideline recommendations and therapeutic availability likely contributed beyond population aging and diagnosis alone. Nonetheless, residual confounding by unmeasured screening cannot be excluded.

The trends observed in the adjuvant HT in patients with higher-risk PCa likely reflects the ongoing efforts to optimize PCa treatment, ensuring that HT is more judiciously applied to cases in which it offers the greatest clinical benefit [32]. These increases also coincide with evolving guideline updates. For example, the 2016 NCCN recommendations incorporated the use of abiraterone and enzalutamide in advanced disease, and the 2018/2020 updates refined HT duration based on clinical risk stratification. Our findings of a post-2016 rise in adjuvant HT align with these guideline shifts [33]. The American Urological Association guidelines recommend radiation therapy alone as an effective management option for low-risk and favorable intermediate-risk PCa [33,34]. However, the rate of primary HT use in patients with lower-risk disease remained relatively stable, ranging from 25 to 28% over the study period [33,34,35]. Taken together, these findings suggest that increases in adjuvant HT use after 2016 likely reflect adoption of NCCN recommendations incorporating novel hormonal agents in advanced disease, while the persistence of primary HT in low-risk disease demonstrates ongoing gaps between practice and AUA guidelines that discourage HT in this setting. The temporal alignment between guideline updates and observed practice patterns supports the validity of our findings, while also highlighting areas where dissemination and implementation of guideline-based care remain incomplete [36,37].

Persistent inappropriate HT use in this group reflects overtreatment, particularly concerning in older men with competing comorbidities, and is consistent with prior reports of continued ADT overuse in the U.S. healthcare system [38]. Our results show a 25.9% rate of primary HT use in lower-risk PCa in 2019, closely aligning with Shahinian′s findings, where rates declined from 38.7% in 2003 to 25.7% in 2005, and lower than Aggarwal′s study (2004–2016), which reported approximately 30% of men with favorable intermediate-risk PCa [39,40]. Although guidelines do not recommend primary HT in low-risk disease, its persistence highlights ongoing challenges in translating evidence into practice. Several factors may explain this pattern, including slow adoption of updated guidelines, patient preference for non-invasive management, and barriers to accessing definitive local therapies such as surgery or radiation [40]. In older men with competing comorbidities, primary HT may also be perceived as a less burdensome alternative, despite limited survival benefit and well-documented risks such as metabolic, cognitive, and cardiovascular complications [41]. Prior studies have similarly noted that overtreatment patterns change slowly, underscoring the importance of provider education, patient counseling, and system-level interventions [42,43]. One might expect wider adoption of robotic-assisted prostatectomy and contemporary radiotherapy techniques to displace primary HT in lower-risk disease; yet use has remained stable at ~25–28%, likely reflecting patient preferences, access limitations, referral patterns, and entrenched practice behaviors [43].

Our study also shows significant associations between patient demographic characteristics and HT use. Older men were more likely to receive HT (43.4%), likely due to their limited eligibility for surgical interventions and a preference for HT as a primary treatment option in managing prostate cancer. This aligns with previous literature suggesting a more combined and aggressive treatment approach in older patients, possibly due to the perception of higher-risk disease or a desire for more immediate disease control [40,41,42,43,44]. Racial disparities were evident, with Hispanic men having the highest overall HT use (40.5%), and primary HT use (31.6%) [44]. These patterns are consistent with prior literature showing that Hispanic men are more likely to receive primary androgen-deprivation therapy and less likely to undergo definitive local treatment than non-Hispanic White men. Contributing factors may include geographic clustering within SEER catchment areas with larger Hispanic populations, socioeconomic disadvantage and insurance status, and limited access to specialty urologic care. Together, these findings point to structural and system-level drivers beyond clinical factors alone and underscore the need for targeted interventions to improve access and promote guideline-concordant care [45].

The categorization of HT use into primary and adjuvant categories is crucial in understanding the nuanced decision-making processes in PCa treatment across different risk groups [44,45]. The increasing trend in adjuvant HT use in patients with higher-risk PCa, particularly in the usage of agonist and antagonist, reflects shifting clinical preferences and possibly evolving guidelines for HT in PCa management [42]. The sharp increase in GnRH antagonist use is particularly notable and likely reflects growing clinical awareness of their lower cardiovascular risk compared with agonists, since antagonists do not cause the initial testosterone surge and have been associated in observational studies and meta-analyses with lower rates of major adverse cardiovascular events compared with agonists, particularly in men with pre-existing cardiovascular disease [41,42,43]. This aligns with emerging evidence and may explain their uptake after 2015. Our study examines more recent patterns of HT use (2010–2019) following reimbursement policy changes, which shifted the focus toward optimizing treatment in high-risk patients while addressing persistent gaps in guideline adherence. Our findings showed a steady increase in adjuvant HT in high-risk prostate cancer (PCa) increasing to 68.1% by 2019. However, primary HT use in low-risk PCa remained relatively stable, ranging from 25% to 28%. This persistent use contrasts with guideline recommendations and emphasizes the ongoing challenge of reducing overtreatment. A prior study examining ADT utilization from 1994 to 2005 found a sharp increase in appropriate adjuvant ADT use (from 40% to 85%). Similarly, inappropriate primary ADT use rose from 30.1% in 1994 to a peak of 44.9% in 2002 but then declined after 2004 to 35.5% in 2005 [39,40,41,42,43].

The differences in HT use trends between our study and previous research may be attributed to shifts in clinical guidelines, evolving treatment strategies, and healthcare policy changes. In the early 2000s, evidence demonstrating the limited survival benefits of primary ADT in low-risk prostate cancer led to a decline in its inappropriate use after 2004. By contrast, our study period reflects a stronger emphasis on risk-adapted treatment strategies, resulting in a more stable trend in primary HT use. Concurrently, advancements in radiation techniques, novel systemic therapies, and improved risk stratification may have contributed to the modest increase in adjuvant HT use for high-risk patients after 2016 [44,45,46].

Policy and reimbursement changes also played a crucial role in shaping HT utilization patterns. The reduction in Medicare reimbursement for GnRH agonists in 2004 led to a rapid decline in inappropriate ADT use, whereas our study period was likely less affected by such financial shifts, contributing to a more stable trend. Additionally, variations in patient demographics, clinical settings, and data sources between the two studies, along with changes in cancer staging and classification criteria, may further explain the discrepancies in reported trends [46,47].

Anti-androgens are frequently used in combination with standard ADT, such as GnRH agonists, especially in the context of metastatic PCa. For instance, the increasing use of newer anti-androgens, such as abiraterone and enzalutamide—approved for metastatic disease—may have contributed to the observed uptick in HT use over time, particularly within the primary cohort [48,49,50,51]. Although we observed a decline in the use of anti-androgens (from 7.9% in 2010 to 5.9% in 2014). Our dataset allowed us to differentiate first-generation anti-androgens (bicalutamide, flutamide, nilutamide) from novel agents, and trends suggest that the late-decade increase was largely driven by the uptake of abiraterone and enzalutamide. This decline may be driven by several factors, including evolving treatment guidelines and shifts in reimbursement policies [50]. Changes in reimbursement policy significantly influenced HT utilization patterns, particularly since anti-androgens are often used in combination with GnRH agonists, especially in higher-risk prostate cancer cases [50,51].

Our results align with Aggarwal et al.’s findings on ADT patterns over time. They observed an increase in HT use among patients with locally advanced PCa, reflecting the upward trend seen in our study, particularly among high-risk PCa patients [40,50,51,52]. Relevant clinical studies showed that men with low and intermediate-risk PCa may not derive benefit from additional HT use, whereas those with advanced metastatic PCa continue to show improved failure-free survival [39]. A recent secondary analysis of the Radiation Therapy Oncology Group (RTOG) randomized trial further supported this trend, showing no significant improvement in the rate of prostate cancer-specific mortality with the addition of 6 months of HT compared to radiation therapy alone in patients with favorable intermediate-risk PCa [51,52,53].

Several limitations need to be considered when interpreting our findings. The study′s focus on men aged ≥66 may limit generalizability to younger populations. This age restriction could potentially overlook trends and outcomes specific to younger populations. Our classification required higher-risk patients to have received radiation within 6 months of diagnosis. Consequently, men with high-risk disease who did not undergo radiation were excluded, which may underestimate overall hormonal therapy use in this group. This approach, however, was intended to align with clinical guideline definitions and to reduce misclassification. The absence of data on BMI and smoking status could influenced the HT patterns. Additionally, SEER-Medicare lacks information on PSA levels, Gleason score and patient preference—all key factors in treatment decision making—which could result in unmeasured confounding. While our study provides insights into the evolving landscape of HT usage in PCa, it primarily reflects trends observed within the SEER-Medicare population and may not fully represent the entire spectrum of patients with PCa.

Despite these limitations, our findings contribute valuable insights into the real-world patterns of HT use in older men with higher-risk PCa, highlighting the HT patterns of care among men with PCa. Our research introduces several innovative aspects that expand the understanding of HT use. First, while previous studies focused on men with intermediate-risk PCa receiving radiation therapy, our study broadens the scope to include men who did not undergo radiation therapy, providing a more inclusive view of HT trends [48,49,50]. Second, our research extends four years beyond Aggarwal’s study, offering a more current assessment of HT trends, reflecting recent shifts in clinical guidelines and treatment preferences. Third, our study examines all HT types, such as GnRH agonists, antagonists, and especially anti-androgens agents, which have rarely been studied as part of HT, allowing for a deeper understanding of therapy adoption across patient groups. Lastly, our study incorporates a broader range of PCa risk categories and stages, from low- to high-risk and localized to advanced disease, offering a more comprehensive view of HT use across diverse patient subgroups.

These results highlight several clinical implications. Primary HT should be avoided in men with low-risk localized disease given its limited survival benefit and significant toxicity, with active surveillance or definitive local therapy favored instead. In contrast, adjuvant HT should be used selectively in high-risk disease treated with radiation, with treatment duration tailored to patient risk and tolerance. The rising use of GnRH antagonists is particularly relevant, as they may offer a more favorable cardiovascular profile than agonists, especially in men with pre-existing cardiovascular disease. At the same time, shared decision making remains essential to ensure patients—particularly older men with multiple comorbidity—are fully informed of the risks and alternatives.

Future studies should aim to address these limitations by incorporating broader patients’ groups to enhance the interpretation of HT utilization trends. In summary, while the increase in adjuvant HT use is encouraging, the persistence of primary HT use highlights the need for targeted efforts to understand inappropriate treatment. Investigating the use of HT requires efforts to reduce inappropriate treatment, improve guideline adherence, and ensure that all patients, regardless of demographic or geographic factors, have access to the most suitable treatment options for their individual cases. This will involve not only clinical reevaluation but also the mitigation of racial and socioeconomic disparities in PCa care. 

## 5. Conclusions

In this large, nationally representative study of U.S. men with prostate cancer, overall hormonal therapy use increased from 2010 to 2019, largely driven by the greater adoption of adjuvant HT in high-risk disease. These trends align with evolving guideline recommendations and the introduction of novel agents, including GnRH antagonists and newer anti-androgens. Despite this progress, approximately one in four men with low-risk prostate cancer continued to receive primary HT, representing persistent inappropriate use.

Significant disparities were also evident, with higher utilization among older, Hispanic patients and men with higher comorbidity score. These findings underscore the need for targeted efforts to reduce overtreatment, improve adherence to guideline-concordant care, and ensure equitable access to appropriate therapies. Future research should further explore drivers of inappropriate HT use and evaluate interventions that promote both optimal treatment decision and equity in prostate cancer care.

## Figures and Tables

**Table 1:** Use of Hormonal Therapy (HT) among Men with a Diagnosis of Incident Prostate Cancer (PCa) from 2010 through 2019, according to Demographic and Clinical Characteristics.

Variable	No. of Subjects	Any HT ^†^	Primary HT in Patients with Lower-Risk PCa ^†^ *n* = 73,840	Adjuvant HT in Patients with Higher-Risk PCa ^†^ *n* = 6769	*p* Value
Men receiving HT		35.1%	27.3%	58.6%	
Year of diagnosis					<0.0001
2010	12,931	31.6%	4261 (25%)	511 (53.6%)	
2011	13,227	31.7%	4382 (22.6%)	462 (52.8%)	
2012	11,648	31.7%	3852 (19.9%)	438 (50.5%)	
2013	12,576	31.6%	4187 (16.9%)	620 (55.2%)	
2014	14,311	32.4%	5028 (22.3%)	520 (52.9%)	
2015	15,517	34.5%	6372 (28.2%)	443 (54.2%)	
2016	16,860	36.2%	6272 (25.2%)	729 (53.8%)	
2017	17,592	38.0%	6683 (26.4%)	664 (59.0%)	
2018	17,520	39.5%	6428 (26.6%)	710 (64.4%)	
2019	17,333	40.2%	6496 (25.9%)	720 (68.1%)	
Age					<0.0001
66–70	45,654	28.0%	15,572 (16.8%)	1455 (54.6%)	
71–75	45,817	33.8%	16,398 (22.2%)	1758 (59.4%)	
76–80	30,747	40.6%	11,965 (29.6%)	1434 (57.8%)	
>80	27,297	43.4%	10,026 (33.8%)	1170 (56.2%)	
Race/Ethnicity ^§^					<0.0001
White	118,613	40.5%	43,136 (23.0%)	4490 (56.7%)	
Black	11,663	34.2%	4426 (30.4%)	418 (55.3%)	
Hispanic	10,876	38.2%	3379 (31.6%)	494 (58.1%)	
Other	8363	37.9%	3020 (27.6%)	415 (63.4%)	
Marital status					0.7538
Married	61,040	35.0%	21,008 (24.5%)	1975 (56.6%)	
Not Married	88,475	35.3%	32,953 (24.4%)	3842 (58.4%)	
Clinical stage					<0.0001
Stage I	5877	11.5%	5272 (6.0%)	-	
Stage II	96,066	32.8%	48,689 (26.4%)	-	
Stage III	18,244	39.2%	-	2298 (59.1%)	
Stage IV	29,328	45.0%	-	3519 (55.9%)	
Tumor grade					<0.0001
Well differentiated	21,078	8.8%	12,684 (4.9%)	-	
Moderately differentiated	43,856	26.4%	17,212 (23.0%)	-	
Poorly differentiated/undifferentiated	48,264	55.5%	-	2301 (73.8%)	
Comorbidity Score					<0.0001
0	45,406	31.4%	16,782 (20.4%)	1492 (61.7%)	
1	42,793	35.3%	15,476 (24.4%)	1641 (59.4%)	
2	27,812	37.1%	10,182 (26.4%)	1155 (57.8%)	
≥3	33,504	38.5%	11,521 (28.6%)	1529 (49.9%)	
Radiation					<0.0001
Yes	34,872	51.3%	-	5817 (57.2%)	
No	114,643	30.3%	53,961 (24.4%)	-	

Among the 149,925 men with a diagnosis of incident PCa from 2010 through 2019, 73,840 patients with lower-risk PCa, and 6769 patients with higher-risk PCa. ^†^ Percentages represent the proportions of men receiving (at least one dose of a gonadotropin-releasing hormone agonist, antagonist, or anti-androgen). Percentages in the “Overall” column are calculated using the full analytic cohort (*n* = 149,925) as the denominator, those in the “Primary HT” column use the lower-risk group (*n* = 73,840) as the denominator, and those in the “Adjuvant HT” column use the higher-risk group (*n* = 6769) as the denominator. Adjuvant HT includes patients who underwent radiation and received HT for the treatment of clinical stage T3 or T4 tumors and high grade (poorly differentiated or undifferentiated). Primary HT includes those who received HT and had clinical stage T1 or T2 tumors, low grade (well-differentiated or moderately differentiated) and did not undergo radiation therapy. ^§^ Race or ethnic group was determined from the SEER database. A hyphen (-) indicates that no patients were represented in that risk category.

**Table 2:** Use of Hormonal Therapy (HT), According to Year of Diagnosis.

Year of Diagnosis	Adjuvant HT in Patients with Higher-Risk PCa			Primary HT in Patients with Lower-Risk PCa ^§^		
	GnRH Agonist	GnRH Antagonist	Anti-Androgen	GnRH Agonist	GnRH Antagonist	Anti-Androgen
2010	281 (8.9%)	13 (2.5%)	173 (8.0%)	1084 (8.6%)	66 (3.6%)	569 (7.9%)
2011	243 (7.8%)	22 (4.2%)	151 (7.0%)	1026 (8.2%)	90 (4.9%)	552 (7.7%)
2012	223 (7.1%)	22 (4.2%)	125 (5.8%)	785 (6.3%)	68 (3.7%)	445 (6.2%)
2013	319 (10.1%)	44 (8.5%)	184 (8.5%)	702 (5.6%)	90 (4.9%)	427 (5.9%)
2014	256 (8.2%)	47 (9.0%)	175 (8.1%)	1095 (8.7%)	179 (9.8%)	655 (9.2%)
2015	240 (7.7%)	38 (7.3%)	175 (8.1%)	1664 (13.3%)	288 (15.8%)	942 (13.2%)
2016	368 (11.8%)	65 (12.5%)	284 (13.1%)	1446 (11.5%)	248 (13.6%)	785 (10.9%)
2017	365 (11.7%)	80 (15.4%)	272 (12.6%)	1600 (12.8%)	282 (15.5%)	908 (12.7%)
2018	411 (13.1%)	85 (16.3%)	285 (13.2%)	1596 (12.7%)	254 (13.9%)	931 (13.0%)
2019	424 (13.6%)	105 (20.2%)	330 (15.3%)	1555 (12.4%)	259 (14.2%)	933 (13.1%)

Patients with higher-risk PCa who received HT and underwent radiation therapy for the treatment of clinical stage T3 or T4 tumors and high grade (poorly differentiated or undifferentiated). ^§^ Patients with lower-risk PCa who received primary HT for the treatment of clinical stage T1 or T2 tumors and low grade (well-differentiated or moderately differentiated) and did not undergo radiation therapy. Percentages for each therapy type and year are calculated using the total number of patients in the corresponding group (higher-risk/adjuvant HT or lower-risk/primary HT) as the denominator. Gonadotropin-releasing hormone (GnRH) agonists work by activating the pituitary gland to produce more follicle-stimulating hormone (FSH) and luteinizing hormone (LH). Gonadotropin-releasing hormone (GnRH) antagonists work by blocking the GnRH receptor, which prevents the release of luteinizing hormone (LH) and follicle-stimulating hormone (FSH) from the pituitary gland. Anti-androgen drugs, also known as androgen receptor antagonists or testosterone blockers, work by blocking the androgen receptor (AR) or reducing androgen production.

**Table 3:** Odds Ratio for the Use of Hormonal Therapy (HT), According to Year of Diagnosis.

Variables		Any HT *n* = 149,925 Odds Ratio			Adjuvant HT in Patients with Higher-Risk PCa (*n* = 6769) * Odds Ratio			Primary HT in Patients with Lower-Risk (*n* = 73,840) Odds Ratio	
	%		95% CI	%		95% CI	%		95% CI
Year of Diagnosis									
2011	31.7%	1.01	0.95–1.05	52.8%	0.87 (990)	0.79–0.96	22.6%	0.97 (244)	0.75–1.25
2012	31.7%	1.00	0.95–1.06		0.74 (768)	0.67–0.82		0.88 (221)	0.68–1.14
2013	31.6%	0.99	0.94–1.05	50.5%	0.61 (706)	0.54–0.67	19.9%	1.06 (342)	0.84–1.35
2014	32.4%	1.04	0.98–1.08		0.86 (1119)	0.77–0.94		0.97 (275)	0.76–1.24
2015	34.5%	1.14	1.08–1.20	55.2%	1.17 (1799)	1.08–1.28	16.9%	1.02 (240)	0.79–1.32
2016	36.2%	1.23	1.16–1.28		1.01 (1583)	0.92–1.11		1.01 (392)	0.80–1.26
2017	38.0%	1.33	1.26–1.39	52.9%	1.07 (1763)	0.98–1.17	22.3%	1.25 (392)	0.98–1.57
2018	39.5%	1.41	1.34–1.48		1.08 (1707)	0.99–1.18		1.56 (457)	1.24–1.97
2019	40.2%	1.45	1.38–1.52	54.2%	1.05 (1684)	0.96–1.15	28.2%	1.84 (490)	1.46–2.33

* Odds ratios were calculated from individual logistic regression models for each group. For all models, the dependent variable was the use of HT within 6 months after diagnosis, and independent variables were the calendar year of diagnosis (as a categorical variable), age at diagnosis, race, SEER region, marital status, score on the comorbidity index, clinical stage and tumor grade. Only results for the year of diagnosis are presented.

## Data Availability

The datasets used to conduct this study are available upon approval of a research protocol from the National Cancer Institute. Instructions for obtaining these data are available at https://healthcaredelivery.cancer.gov/seermedicare/obtain/ (accessed on 7 January 2024).

## Appendix A

Flow chart of patients who met the inclusion/exclusion criteria for the study.

## Appendix B

**HT (NDC and HCPCS) Codes**

**Drug Name**	**NDC Code**	**HCPCS Code**
**Agonists**		
Leuprolide (Lupron, Eligard)	00074-3642, 00074-3473, 00074-3683, 00185-7400, 00781-4003, 47335-0936, 55150-0478, 62935-0223, 62935-0303, 62935-0453, 62935-0753, 69448-0014, 72664-0611	J1950, J1952, J9217, J9218, J9219
Goserelin (Zoladex)	50090-2027, 50090-3466, 70720-0950, 70720-0951	J9202
Triptorelin (Trelstar)	00023-5902, 00023-5904, 00023-5906	J3315
Histrelin	67979-0501, 67979-0401	J1675, J9225, J9226
**Antagonists**		
Degarelix	55566-8303, 55566-8403	J9155
Relugolix	72974-0120	
**Antiandrogens**		
*Old generation drugs*		
Flutamide	69097-0915	
Nilutamide	59212-0111, 62559-0173, 66993-0212, 82454-0212	
Bicalutamide	00904-6019, 16714-0571, 16714-0816, 16729-0023, 47335-0485, 54868-4503, 54868-6133, 60429-0177, 60505-2642, 62559-0680, 62559-0890, 63629-5321, 63672-0005, 65841-0613, 67253-0191, 68382-0224	
*New generation drugs*		
Enzalutamide	00469-0125	
Apalutamide	59676-0600	
Darolutamide	50419-0395	
*Abiraterone (CYP17 inhibitor)*	00093-1125, 00143-9597, 00378-6920, 00904-6948, 16714-0963, 42291-0024, 42291-0073, 57894-0150, 57894-0184	

**ICD 9 and ICD 10 Codes**

**Treatment**	**ICD-9, ICD-10 or HCPCS/CPT Code**
Orchiectomy	(CPT codes 54520) **ICD-9:** 62.4, V45.77 **ICD-10:** Z90.79
Radiation Therapy	External beam radiotherapy (CPT codes 77400–77416, 77420, 77425, 77430), Brachytherapy (CPT codes 77776–77778), **ICD-9:** 92.21–92.27, 92.3 **ICD-10:** Z51.0 DVY07ZZ HCPCS/CPT: 77401–77416, 77418, 77520, 77522–77525, 55875, 77373
prostatectomy	**ICD-9:** 60.3, 60.4, 60.5, V45.77 **ICD-10:** Z90.79 HCPCS/CPT: 55801, 55810, 55812, 55815, 55840, 55842, 55845, 55860, 55862, 55865, 55866, 52601, 52612, 52614
**Demographic characteristics and comorbidities**	
Age	NAACCR Item Number 230
Race/Ethnicity	Non-Hispanics white, Non-Hispanics Black, Hispanics, others
Congestive heart failure	**ICD-9** 398.91, 402.01, 402.11, 402.91, 404.01, 404.03, 404.11, 404.13, 404.91, 404.93, 428.0–428.9 **ICD-10** (I50)
Valvular disease	**ICD-9** 093.20–093.24, 394.0–397.1, 397.9, 424.0–424.99, 746.3–746.6, V42.2, V43.3 **ICD-10** (I35)
Pulmonary circulation disorders	**ICD-9** 415.11–415.19, 416.0–416.9, 417.9 (491–496) **ICD-10** (I26–I27)
Peripheral vascular disorders	**ICD-9** 440–440.9, 441.00–441.9, 442.0–442.9, 443.1–443.9, 444.21–444.22, 447.1,449, 557.1, 557.9, V43.4 (443) **ICD-10** (I73)
Lymphoma	**ICD-9** 200.00–202.38, 202.50–203.01, 203.02–203.82, 203.8–203.81, 238.6, 273.3 **ICD-10** (C85)
Diabetes mellitus	**ICD-9** 249.00–249.31, 250.00–250.33, 648.00–648.04, 249.40–249.91, 250.40–250.93, 775.1 (250) **ICD-10** (E08–E13)
Hypertension	**ICD-9** 401.1, 401.9, 642.00–642.04, 401.0, 402.00–405.99, 437.2, 642.10–642.24, 642.70–642.94 (401–405) **ICD-10** (I10)
Hypothyroidism	**ICD-9** 243–244.2, 244.8, 244.9 **ICD-10** E03
Hyperlipidemia	**ICD-9** (272) **ICD-10** (E78)
Chronic kidney disease	**ICD-9** 403.01, 403.11, 403.91, 404.02, 404.03, 404.12, 404.13, 404.92, 404.93, 585.3, 585.4, 585.5, 585.6, 585.9, 586, V42.0, V45.1, V45.11, V45.12, V56.0-V56.32, V56.8 **ICD-10** N18, N17, N19
Chronic liver disease	**ICD-9** 070.22, 070.23, 070.32, 070.33, 070.44, 070.54, 456.0, 456.1, 456.20, 456.21, 571.0, 571.2, 571.3, 571.40–571.49, 571.5, 571.6, 571.8, 571.9, 572.3, 572.8, 573.5, V42.7 **ICD-10** (K76, K75)
Chronic pulmonary disease	**ICD-9** 490–492.8, 493.00–493.92, 494–494.1, 495.0–505, 506.4, **ICD-10** (J41–J44, I28)
Chronic peptic ulcer	**ICD-9** 531.41, 531.51, 531.61, 531.70, 531.71, 531.91, 532.41, 532.51, 532.61, 532.70, 532.71, 532.91, 533.41, 533.51, 533.61, 533.70, 533.71, 533.91, 534.41, 534.51, 534.61, 534.70, 534.71, 534.91 **ICD-10** K27
Cerebral vascular accident	**ICD-9** (430–438) **ICD-10** (I60–I67)
Neurological disorders	**ICD-9** 330.1–331.9, 332.0, 333.4, 333.5, 333.71, 333.72, 333.79, 333.85, 333.94, 334.0–335.9, 338.0, 340, 341.1–341.9, 345.00–345.11, 345.2–345.3, 345.40–345.91, 347.00–347.01, 347.10–347.11, 649.40–649.44, 768.7, 768.70, 768.71, 768.72, 780.3, 780.31, 780.32, 780.33, 780.39, 780.97, 784.3 **ICD-10** G20, G35, G40–G41, G60–G64, G80–G83
HIV and Aids	**ICD-9** 042–044.9 **ICD-10** B20, Z21, Z83
Metastatic cancer	**ICD-9** 196.0–199.1, 209.70, 209.71, 209.72, 209.73, 209.74, 209.75, 209.79, 789.51 **ICD-10** C77, C80, C7B
Solid tumor without metastasis	**ICD-9** 140.0–172.9, 174.0–175.9, 179–195.8, 209.00–209.24, 209.25–209.3, 209.30–209.36, 258.01–258.03 **ICD-10** C00, C43, C50, C55, C76
Rheumatoid arthritis/collagen vascular diseases	**ICD-9** 701.0, 710.0–710.9, 714.0–714.9, 720.0–720.9, 725 **ICD-10** M05–M06, M32, M34, M35.3
Coagulation deficiency	**ICD-9** 286.0–286.9, 287.1, 287.3–287.5, 289.84, 649.30–649.34 **ICD-10** D68
Obesity	**ICD-9** 278.0, 278.00, 278.01, 278.03, 649.10–649.14, 793.91, V85.30-V85.39, V85.41-V85.45, V85.54 **ICD-10** E66
Weight loss	**ICD-9** 260–263.9, 783.21, 783.22 **ICD-10** R63
Fluid and electrolyte disorders	**ICD-9** 276.0–276.9 **ICD-10** E87
Blood loss anemia	**ICD-9** 280.0, 648.20–648.24 **ICD-10** D50
Deficiency anemias	**ICD-9** 280.1–281.9, 285.21–285.29, 285.9 **ICD-10** D51, D52, D55, D53
Alcohol abuse	**ICD-9** 291.0–291.3, 291.5, 291.8, 291.81, 291.82, 291.89, 291.9, 303.00–303.93, 305.00–305.03 **ICD-10** F10
Drug abuse	**ICD-9** 292.0, 292.82–292.89, 292.9, 304.00–304.93, 305.20–305.93, 648.30–648.34 **ICD-10** Z71, F19, F11, F55, F15
Psychoses	**ICD-9** 295.00–298.9, 299.10, 299.11 **ICD-10** F29, F23, F53
Depression	**ICD-9** 300.4, 301.12, 309.0, 309.1, 311 **ICD-10** F32
